# Evaluation of Periodontal Infrabony Defect Topography via CBCT and Comparisons with Direct Intrasurgical Measurements

**DOI:** 10.3390/bioengineering12070780

**Published:** 2025-07-18

**Authors:** Tiffany See Nok Chen, Nicholas David Sung, Melissa Rachel Fok, Mihai Tarce, Kanoknadda Tavedhikul, Georgios Pelekos

**Affiliations:** 1 Division of Periodontology and Implant Dentistry, Faculty of Dentistry, Prince Philip Dental Hospital, The University of Hong Kong, 34 Hospital Road, Hong Kong SAR, China; tiffc092@connect.hku.hk (T.S.N.C.); nicksung@connect.hku.hk (N.D.S.); melfok@hku.hk (M.R.F.); mihai@hku.hk (M.T.); 2 Department of Periodontology, Faculty of Dentistry, Chulalongkorn University, Bangkok 10330, Thailand; kanok.kanok@gmail.com

**Keywords:** infrabony defects, cone beam computed tomography, direct intrasurgical measurements

## Abstract

**Background:** Two-dimensional periapical radiographs (PAs) only offer limited information regarding three-dimensional periodontal infrabony defects. In contrast, cone beam computed tomography (CBCT) enables visualization of the entire defect morphology. This study aimed to evaluate the agreement between CBCT and direct intrasurgical measurements (ISs) regarding the characteristics of infrabony defects, including measurements of defect depth, width, the type of defect (one-wall, two-wall, three-wall), and defect extension. **Methods:** Intrasurgical and radiographic assessments were performed by two calibrated examiners on 26 infrabony defects in 17 patients who underwent periodontal surgery. The defect depth, width, type, and extension were compared between intrasurgical observations and PA or CBCT findings. The CBCT assessment was performed mainly using axial reconstructions. Angle measurements were compared between CBCT and PAs. **Results:** The mean differences between CBCT and intrasurgical measurements were −0.11 ± 0.49 mm for depth and −0.07 ± 0.41 mm for width, with no significant differences. The ICC values were 0.938 and 0.923 for depth and width, respectively. The mean difference in width between PAs and ISs was significantly different (−0.36 ± 0.73 mm; *p* = 0.002). CBCT demonstrated high agreement with intrasurgical observations for defect type (κ = 0.819) and defect extension (κ = 0.855), while lower agreements were found for PAs. **Conclusions:** CBCT is a valid assessment modality for infrabony defects. It demonstrated strong agreement with ISs—as the gold standard—for depth and width measurements, and its agreement with ISs regarding defect type and extension appeared to surpass that of PAs.

## 1. Introduction

Periodontal bone loss can present a horizontal or vertical pattern and defects can be classified into one-wall, two-wall, and three-wall defects—or, in many cases, combination defects—with regard to the number of residual bone walls around the defect [1,2]. As the presence of infrabony defects is associated with increased bone loss and frequency of tooth loss [3], the adequate diagnosis and treatment of infrabony defects are crucial. The success of regenerative procedures depends on the characteristics of the defect—greater depth, narrower angles (<37°), and more residual bone walls predict better outcomes [3]. Surgical planning, including flap design, also relies on the detailed assessment of these defect features [4]. A systematic review and meta-analysis has compared different types of flaps in access flap surgery for infrabony defects [5]. Minimally invasive techniques are now favored due to their improved wound stability, greater clinical attachment level (CAL) gain, and patient-centered outcomes, making the precise assessment of infrabony topography essential.

At present, intrasurgical evaluation is regarded as the gold standard for assessing defects. Among presurgical assessment modalities, transgingival bone sounding has demonstrated reliable accuracy in determining interproximal bone levels [6]; however, this method requires local anesthesia and is traumatic to the soft tissues. Intraoral radiography remains the most commonly employed imaging technique, enabling high-resolution visualization of the lamina dura [7]. Nonetheless, its diagnostic utility is constrained by anatomical superimposition, projection errors, and an inability to differentiate between buccal and lingual cortical plates or to detect osseous dehiscence and fenestration [8,9].

CBCT is an alternative modality for assessing infrabony defects, which has been shown to agree well with the gold standard. A systematic review [10] has reported that, for vertical and horizontal bone loss, CBCT demonstrated an accuracy of 58–93% among 4 out of 6 included studies; another study [11] reported no difference between the gold standard and CBCT measurements of bone loss. CBCT enables accurate linear assessment and visualization of defect morphology [12,13,14], and has also been proposed for the evaluation of periodontal regenerative outcomes [15,16,17].

The published literature demonstrating the accuracy of CBCT in infrabony defect assessments primarily consists of in vitro studies, with the gold standard being artificially created defects on animal’s mandibles [18,19,20], or artificial [8,18,21,22,23,24,25,26,27] or naturally occurring defects [28] in human dry skulls or cadavers. These models have inherent limitations, including the anatomical differences between animal and human skulls (e.g., the thickness and density of alveolar bone) [20,29]. Furthermore, artificially created defects lack the characteristic irregular borders or complex defect morphology of naturally occurring periodontal lesions [13].

Only a limited number of studies have compared the accuracy of CBCT in assessing infrabony defects specifically, taking surgical measurements as the gold standard. One study involving 100 defects reported a strong correlation of 0.988 between CBCT and intrasurgical measurements [30], while another found no significant differences across varying CBCT section thicknesses [31]. Several studies [32,33,34] have further demonstrated a higher correlation between CBCT and surgical measurements than between intraoral radiographs and surgical measurements. A randomized controlled trial [15] has also confirmed CBCT’s superior accuracy in evaluating regenerative outcomes, although it slightly underestimated the cementoenamel junction (CEJ) to defect base by 0.5–0.9 mm with respect to surgical measurements. More recent research has demonstrated the accuracy of CBCT in measuring the mesiodistal defect width and its ability to assess buccolingual dimensions, although it was shown to offer no advantage over periapical radiographs for vertical measurements [35]. In contrast, one study reported that the measurements on CBCT did not agree with intrasurgical measurements [36], although periodontal bone loss was measured at six sites per tooth rather than evaluating infrabony defects.

Several ex vivo studies have investigated the classification of defect morphology using CBCT. Bayat et al. [20] have reported that CBCT was significantly superior for detecting three-wall defects, fenestrations, and dehiscence when compared to intraoral radiographs. Similarly, a study using pig mandibles [19] reported more precise analysis and improved detection in the orovestibular dimension with CBCT. Other studies [22,23] have also concluded that CBCT allows for better morphological description and classification of defects than digital radiography. Additionally, it has been suggested that CBCT was superior in the evaluation of the number of defect walls and treatment among three periodontists, when compared to intraoral radiographs [37]; however, comparisons were only made between assessments using CBCT and intraoral radiographs.

Of the in vivo studies mentioned above, most focused on linear measurements and did not report the accuracy of CBCT in other aspects of defect assessment. Only one study [35] reported on the residual bony walls of the defects in their sample, but no detailed comparisons with the CBCT observations or analysis were made. There remains a gap in the literature regarding the reliability of CBCT in assessing other morphological features of infrabony defects that are important for effective treatment planning.

This study aims to evaluate the potential of CBCT in assessing periodontal infrabony defect characteristics—including their depth, width, defect type, and extension—through comparing CBCT measurements and observations with intrasurgical measurements (ISs) during periodontal surgery, considered as the gold standard. If CBCT can provide clinicians with reliable data reflecting the essential characteristics of defects—thus giving a complete picture of the defect morphology and topography—it can facilitate treatment decisions and presurgical planning.

## 2. Materials and Methods

### 2.1. Study Design and Subject Recruitment

This was a cross-sectional study. Consenting patients aged 18 years or older who were planned to receive periodontal surgery at The Prince Philip Dental Hospital (PPDH) and/or the Institute for Advanced Dentistry-Multi-Specialty Clinic, The University of Hong Kong (HKU IAD-MSC) by periodontal staff or residents were selected.

All patients were diagnosed with Stage III or Stage IV periodontitis and had no contraindications for radiography or periodontal surgery. They possessed at least one periodontal infrabony defect indicated for surgical intervention after non-surgical periodontal therapy (NSPT). They had also already acquired (or were indicated for) a CBCT record after non-surgical periodontal therapy, prior to the planned periodontal or implant surgery. CBCT images with >200 μm voxel size or of inadequate diagnostic quality for infrabony defect assessment were excluded. Defects were excluded if there were associations with (1) furcation involvement; (2) metallic restorations, root-filling materials, or dental implants at neighboring sites that produced artifacts on the CBCT image, affecting the assessment of infrabony defects; and (3) a tooth suffering from secondary occlusal trauma with apparent widening of periodontal ligament space on radiographic images.

### 2.2. Collection of Presurgical Data

Baseline and presurgical (i.e., after non-surgical therapy) periapical radiographs, as well as periodontal charts of the site—which included recession, probing pocket depths, and bleeding on probing at six sites per tooth—were collected from the assigned operators. Presurgical photos of the areas of interest were also obtained.

### 2.3. Radiographic Examination

All radiographic images were obtained at PPDH or HKU IAD-MSC by periodontal residents or staff in the Faculty of Dentistry, HKU.

- Parallel Periapical Radiographs (PAs)

Digital periapical radiographs were taken with the Morita Veraview iX unit (Morita, Saitama, Japan) and photostimulable phosphor plates (Dürr Dental, Baden-Württemberg, Germany), using a setting of 60 kV, 7 mA, and an exposure time of 0.16 s, 0.20 s, 0.25, 0.32, or 0.40 s, depending on the region of interest. Size 2 film was used for the posterior teeth, while Size 0 film was chosen for the anterior teeth to obtain a parallel image, allowing for observation of the mesial and distal alveolar bone of the teeth. The paralleling technique was applied with the help of a film holder device with an aiming ring (Rinn XCP, Dentsply, Elgin, IL, USA). The radiographs were scanned and digitized with the Durr VistaScan Mini view machine (Dürr Dental, Baden-Württemberg, Germany).

- CBCT

All CBCT images were obtained using either the Morita Veraview X800 (Morita, Saitama, Japan) or Planmeca ProMax (Planmeca, Helsinki, Finland) using a setting of 90–120 kV, 5–6.3 mAs, and an exposure time of 8–10 s, within 7 months prior to the surgery. Notably, no active periodontal treatments were performed during this period. The FOV ranged from 8.0 × 8.0 cm to 10.0 × 10.0 cm and the voxel size was 125–200 μm. Image data were imported into the Romexis viewing software (Romexis 3.2, Planmeca, Helsinki, Finland) for reconstruction and viewing.

### 2.4. Measurements

Two trained and calibrated examiners (T.C. and N.S.) who were final-year students in the Master of Dental Surgery (Periodontology) program were involved in the intrasurgical data collection and in the measurement and evaluation of the periapical radiographs and CBCT. For calibration of intrasurgical measurements, both examiners measured the first five cases. PA and CBCT images were viewed on a standardized computer monitor with a 1920 × 1080 screen resolution. The Romexis viewing software (Romexis 3.2, Planmeca, Helsinki, Finland) was used to display the PA and CBCT images, and the measurement tools in the software were used to perform measurements. To ensure the reliability of measurements, linear parameters for each modality were measured twice by each examiner for all the defects in the sample and at least 3 days apart, with the cases presented in a randomized order. Measurements between modalities were performed at least one week apart. The examiners were blinded to the measurements using other modalities and to the ISs of the randomized cases while assessing the radiographs. They were allowed to adjust the image and magnification of the radiographic images freely.

- Intrasurgical Measurements

**Figure 1:**
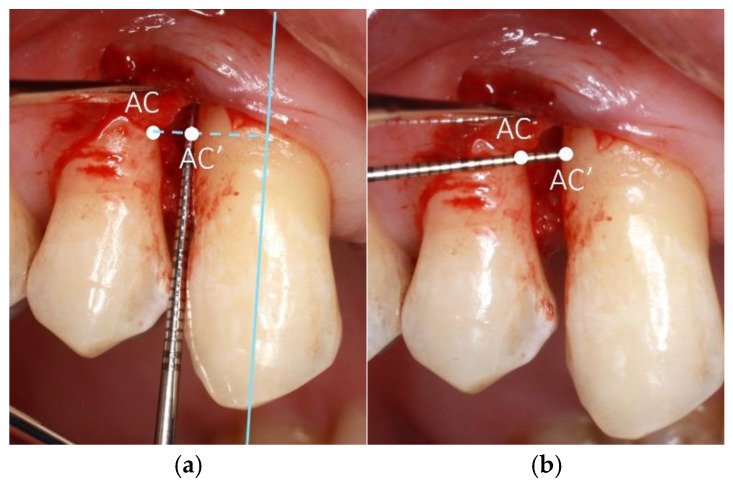
(**a**) (**Left**). Example of intrasurgical measurement of defect depth. AC = most coronal point of adjacent bone crest; AC’ = the intersection of the root surface with a line joining perpendicular from AC to the long axis of the tooth. Depth of defect is measured from the base of the defect to AC’. (**b**) (**Right**). Example of intrasurgical measurement of defect width. AC = most coronal point of adjacent bone crest; AC’ = the intersection of the root surface with a line joining perpendicular from AC to the long axis of the tooth. Width is measured from AC to AC’.

After degranulation of the infrabony defects, measurements were taken to the nearest 0.5 mm by a calibrated examiner using a 15 mm Cortellini Periodontic Probe (HuFriedy, Chicago, IL, USA) with markings at 0.5 mm increments. The examiner was blinded to the periodontal charts and the radiographic information. The following data were obtained:

Defect depth: Measured by locating the most coronal point of the interproximal alveolar crest (AC) of the adjacent tooth and the base of the defect (BD). The buccolingual/buccopalatal limits of the AC location were the buccal and lingual/palatal line angles of the root. The probe was placed along the root surface of the defect-involved tooth to the base of the defect (BD). The point AC was joined to the root surface of the defect-involved tooth by an imaginary line perpendicular to the long axis of the tooth. For multi-rooted teeth, the long axis of the root was considered. This was aided by another probe placed horizontally during intrasurgical measurements. The intersection point of this line and the root surface was named AC’ (Figure 1a). The depth measurement was taken from AC to BD.

Defect width: The widest width of the defect was measured by locating the point of the adjacent bone crest with the greatest distance from the defect-involved root surface. The measurement was taken from this point to the root, along a line perpendicular to the long axis, as well as perpendicular to the tangent of the root surface (Figure 1b).

Defect type: The defect morphology was classified into one-wall, two-wall, or three-wall based on the examiner’s judgment of which was the predominant component of the defect (i.e., contributed to the largest defect depth). The classification was based on definitions described by Goldman and Cohen (1958) [1]. Interproximal craters were considered two-wall defects.

Defect extension (around root surfaces): A defect was considered to have extended to a root surface if the defect area passed the line angle of the root adjacent to the root surface. Whether the defect involved the mesial, distal, buccal, and/or lingual/palatal surfaces was noted.

- Parallel Periapical Radiographs (PAs)

**Figure 2:**
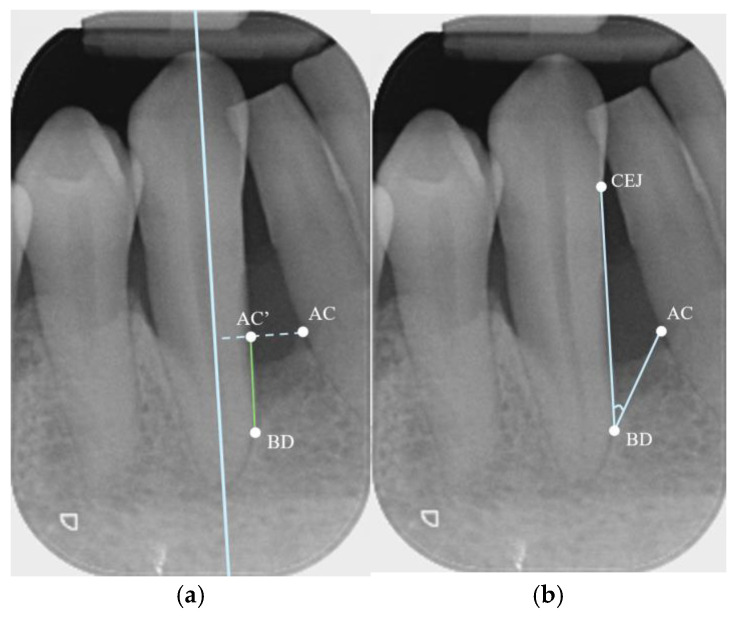
(**a**) (**Left**). Example of depth and width measurements on a PA. AC = most coronal point of adjacent bone crest; AC’ = the intersection of the root surface with a line joining perpendicular from AC to the long axis of the tooth; BD = base of defect. Depth of defect is measured from AC’ to BD; width is measured from AC to AC’. (**b**) (**Right**). Example of angle measurement on a PA. CEJ = cementoenamel junction; AC = most coronal point of adjacent bone crest; BD = base of defect. Defect angle is the angle between the lines CEJ-BD and AC-BD.

Defect Depth: Depth was measured based on the method described by Eickholz et al. [38]. A line was drawn along the long axis of the tooth (or root, for multi-rooted teeth). A second line was drawn from the most coronal point of the alveolar crest (AC) towards and perpendicular to the first line. The intersection between the second line and the root surface contour was labeled AC’. Then, BD was defined as the most coronal point at which the periodontal ligament space still retained its uniform width [39]. The depth of the defect was measured as the distance from AC’ to BD (Figure 2a).

Defect width: The width was measured from AC to the root surface of the defect-involved tooth, along a line perpendicular to the long axis of the tooth/root (from AC to AC’) [38] (Figure 2a).

Defect angle: The angle was measured according to the method described by Steffensen and Webert [40]. A line was drawn from AC to BD, as well as another line from BD to the CEJ of the defect-involved tooth, and the angle between these two lines was measured (Figure 2b). If a restorative material was present at the CEJ, the apical margin of the restoration was taken as the landmark.

Defect type and extension: These were defined as described in the intrasurgical measurement section. The examiners were provided with the baseline and presurgical clinical chart data to make judgments on these parameters. The examiners discussed and reached an agreement for cases with discrepancies.

- CBCT

**Figure 3:**
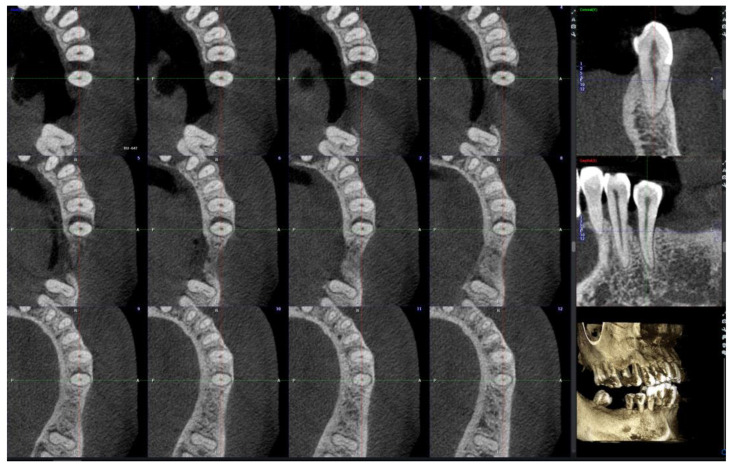
Example of CBCT slices in the coronal and sagittal views (**right column**), as well as axial slices at a distance of 0.5 mm apart, from the coronal (slice 1, **upper left**) to the apical direction (slice 12, **lower right**). The infrabony defect is visible on 7 axial slices (from slice 3 to 9).

A slice distance of 0.5 mm and a slice thickness of 0.2 mm were used for assessment of the CBCT. Examiners were free to move the X-, Y- and Z-axes to evaluate the 3-dimensional data. The Z-axis was aligned with the long axis of the tooth (long axis of the root for multirooted teeth). Coronal, sagittal, and axial images were visible simultaneously on the monitor. Multiple axial cuts were concurrently displayed (Figure 3). Parameters were recorded using the following methods:

Defect depth—Viewing from the axial reconstruction, the number of axial slices where the defect was within view was counted (i.e., from the slice on which the most coronal point of the interproximal alveolar crest of the adjacent tooth (AC) was located to the slice where the point BD was located). The defect depth was calculated using the following formula: (Number of axial slices − 1) × slice distance (0.5 mm) + slice thickness (0.2 mm).

Defect width—The widest width was located and measured on the axial slices, with a line drawn from point AC of the adjacent tooth to the root surface, perpendicular to the long axis of the tooth/root, and perpendicular to a tangent to the root surface. Measurements were corrected to the nearest 0.1 mm.

**Figure 4:**
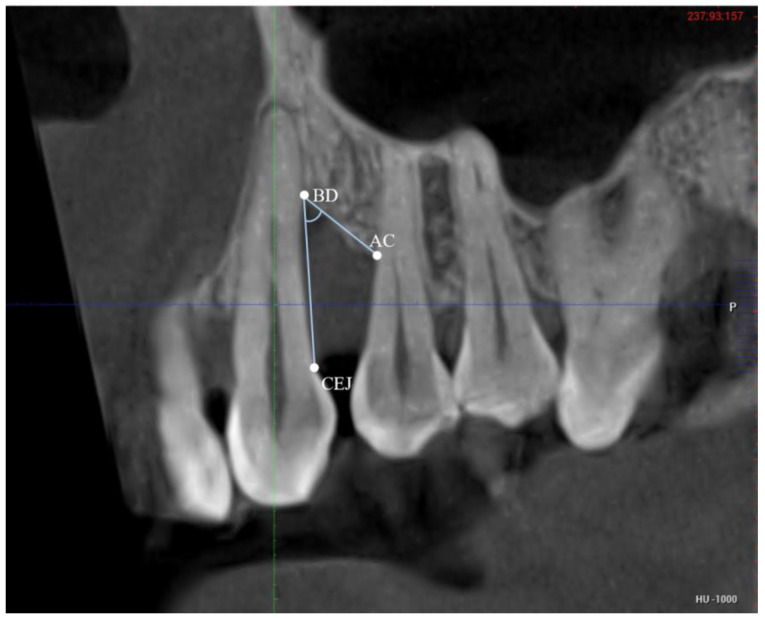
Example of a CBCT cut used for angle measurement. CEJ = cementoenamel junction; AC = most coronal point of adjacent bone crest; BD = base of defect. The defect angle is the angle between the lines CEJ-BD and AC-BD.

Defect angle—The angle was measured on a view (coronal/sagittal) that showed the mesial and distal bone levels of the teeth (Figure 4). First, this cut was produced by aligning with the center points of the roots of the defect-involved tooth and the adjacent tooth. The point AC was located from the axial slices, and this point was marked on the coronal/sagittal view by translating the vertical axis in the buccolingual/buccopalatal direction until the point AC was in view. This slice was then translated in the same way until the BD was in view. On that slice, the angle was measured between the marked point AC, the point BD, and the CEJ (or restorative margin). In this way, the angle was measured on a plane parallel to the line passing through the centers of the roots and perpendicular to the long axis, while concurrently considering the levels of AC and BD (even though they might not be visible on the same slices). Measurements were corrected to the nearest 0.1 degree.

Defect type and extension—These were recorded in accordance with the definitions used in the intrasurgical data collection, and were determined from the axial views.

### 2.5. Sample Size and Statistical Analysis

The sample size was calculated for a two-tailed paired *t*-test. Based on the previous studies, the anticipated standard deviation of the difference in defect depth is 0.6 mm [34]. Using the G*power software 3.1.9.6 (Universität Kiel, Germany), a sample size of 25 was determined to be required to have 95% power and for the limits of the two-sided 95% confidence interval to exclude the mean difference of more than 0.5 (implied effect size d of 0.5/0.6 = 0.83), assuming no difference between the IS and CBCT measurements.

The data were analyzed using the SPSS 27.0 software (IBM corp, Armonk, NY, USA). Intra-examiner and inter-examiner agreements were assessed with the intraclass correlation coefficient (ICC) and Cohens’ kappa statistics. ICCs were computed between intrasurgical measurements and the other two modalities investigated. The ICCs for defect angle were computed between PAs and CBCT. Mean differences between intrasurgical measurements and the CBCT- and PA-based measurements were analyzed using the paired-T test. Cohen’s kappa was used to analyze the agreement of the two modalities compared to intrasurgical observations for defect type and extension. The McNemar test was used to determine whether there were significant differences between the percentage of agreement of CBCT and that of PAs. The significance level in all tests was set at 0.05.

## 3. Results

### 3.1. Demographics

A total of 17 patients with 26 defects were included in the study. Patients were between 26 and 69 years of age. A total of 3 patients were recruited from PPDH, while 14 were recruited from HKU IAD-MSC. The demographics and clinical characteristics are presented in Table A1 (Appendix A), and a summary of the statistics for linear measurements and angles is given in Table A2 (Appendix A).

### 3.2. Intra-Examiner and Inter-Examiner Agreement

The ICCs for intra-examiner and inter-examiner agreement for single measures are reported in Table A3 (Appendix A). Extremely high intra-examiner reliability, with an intraclass correlation coefficient (ICC) greater than 0.9, was demonstrated for all variables and both observers. Complete agreement was observed among observers for depth measurements on CBCT, which was mainly due to the measurement method selected. Inter-examiner ICCs were also >0.9 except for PA angle measurements. Cohen’s kappa was computed for defect type and defect extension between observers (Table A4, Appendix A), demonstrating high agreement for CBCT in both variables, as well as substantial but lower agreement for PAs. To address the differences in the determination of defect type and extension between examiners, a consensus was reached and incorporated into the data analysis.

### 3.3. Agreement Between ISs and CBCT or PAs for Linear Measurements

The ICCs between ISs and CBCT or PAs are provided in Table 1. The ICCs for IS versus CBCT measurements were 0.938 for depth and 0.923 for width, representing excellent agreement. Good reliability was also found for PAs (ICC = 0.790 for depth, 0.704 for width), although the figures were lower than those for CBCT.

The paired *t*-test revealed mean differences of −0.11 ± 0.49 mm (95% CI: −0.31–0.09) and −0.07 ± 0.41 mm (95% CI: −0.23–0.01) between CBCT and ISs for defect depth and width respectively, and the difference was not significant (*p* > 0.05) (Table 2). Meanwhile, the depth and width measurements of PAs compared to ISs were 0.15 ± 0.95 mm (95% CI: −0.23–0.53) and −0.36 ± −0.73 mm (95% CI: −0.65–0.06) (*p* = 0.002), respectively, and significant differences were found between the PA and IS width measurements.

### 3.4. Agreement Between CBCT and PAs for Angle

For angle measurements, the intraclass correlation coefficient (ICC) between CBCT and PA was 0.894 (95% CI: 0.779–0.951), indicating good agreement.

### 3.5. Agreement Between IS and PA or CBCT Observations of Defect Type and Extension

The kappa values for defect type and defect extension between the IS gold standard and PAs or CBCT are illustrated in Table 3. High agreement was found between CBCT and IS observations for both defect type (κ = 0.819) and defect extension (κ = 0.855). In contrast, poor agreement was demonstrated regarding the PA defect assessment, with kappa values of less than 0.4; in particular, only 46% of defect types and 54% of defect extensions were in agreement with the IS observations. The differences in the percentages of total agreement with IS observations for both defect types and extensions, when comparing CBCT and PAs, were indicated by McNemar’s test to be significant (*p* = 0.007 and *p* = 0.012, respectively).

Further comparisons were made between the results regarding defect extension. Of these 14 selected cases, CBCT showed an error in detecting the extension in three cases. Extensions to the lingual surface and buccal surface were undetected in two cases, respectively. For one mesial defect, lingual and distal extensions were observed on CBCT but not intra-surgically. CBCT successfully detected the rest of the extensions to buccal (4 out of 5), lingual or palatal (10 out of 11), and the inter-proximal surface opposite to the main defect side (5 out of 5). On the other hand, 4 out of 5 buccal extensions, 9 out of 11 lingual/palatal extensions, and 4 out of 5 opposite interproximal surfaces could not be observed on PAs. One case showed an incorrect identification between the extension to the buccal or lingual side of the root. In another case, the extension to the palatal and the opposite interproximal surface was incorrectly detected.

## 4. Discussion

The aim of this study was to evaluate the degree of agreement between CBCT and intrasurgical infrabony defect characteristics, including defect depth, width, type, and extension. Strong agreement was found between CBCT and ISs for all characteristics. PA and CBCT measurements of angle were additionally compared, also demonstrating good agreement.

### 4.1. Linear Measurements

The highest ICCs were found between CBCT and ISs for depth and width. In another study comparing the accuracy of CBCT versus intraoral radiographs for periodontal bone defect measurements in dry human mandibles [29], it was reported that the ICC between CBCT and gold standard measurements was 0.93, while that between intraoral radiographs and gold standard was 0.78. The current study found comparable ICCs—0.938 between CBCT and ISs, and 0.790 between PAs and ISs—for depth measurements. For defect width, the ICC between CBCT and ISs was also high, at 0.923, while that between PAs and ISs was comparatively lower, at 0.704. This could have been due to the definition used for width measurements in this study; namely, the greatest distance from the bone crest to the root surface, which may not be precisely at the mid-interproximal area.

The mean error for depth when compared to ISs was found to be −0.11 ± 0.49 mm for CBCT, with a *p*-value of > 0.05 indicating no significant difference from ISs. In the existing literature, a range of mean differences have been described. Compared to an in-vitro study [8], which reported a 0.41 mm mean CBCT measurement error for height and a 1 mm error for width, the mean errors found in our present study were much lower. An in vivo study [35] has reported a CBCT mean error of 0.63 mm for depth of defect. One study [34] found a CBCT mean difference of 0.53 mm from the gold standard while, for intraoral radiographs, the mean error was 0.56 mm. These findings were still considerably larger than the present results. Meanwhile, another study found a more comparable result to this present study, reporting a mean error of 0.07 mm [33]. For PA depth measurements, the mean difference was found to be 0.15 mm in this study, which is also smaller than the 1.4 mm mean difference reported by Zybutz et al. [6]. However, it should be noted that the positive or negative values may have canceled each other out in the paired *t*-test analysis, leading to a smaller overall mean error.

For width measurements, the present study found a higher mean difference of −0.36 ± 0.73 mm for PAs compared to ISs, with significant differences from ISs (*p* < 0.05). However, the mean error in width measured on CBCT was only −0.07 ± 0.41 mm. One study [35] has reported a similar CBCT mean error of −0.17 mm for mesiodistal width. As mentioned previously, the width measurement methodology may have contributed to this discrepancy in results between CBCT and PAs.

### 4.2. Agreement in Defect Type and Extension

Comparing the determination of defect type and extension to root surfaces, CBCT demonstrated much better agreement with ISs than PAs, with the differences in percentage of total agreement being statistically significant. This was an expected outcome, as the three-dimensional topography of the defect can be visualized on CBCT. Errors in determining the predominant defect configuration (one-wall, two-wall, three-wall) could be due to inaccurate judgment regarding the demarcations of bony walls. In terms of defect extension, it is interesting to note that, in one case, a narrow extension to the palatal and distal side of the root surface was observed on CBCT, while there was no apparent defect intrasurgically. The CBCT data were obtained only one and half months before the surgery, so it was unlikely that this discrepancy was caused by bone fill in such a short time. One explanation for this finding is that the observed narrow radiolucent area was filled with attachment or less mineralized bone, which appeared radiolucent on imaging but was not detected as a defect intrasurgically. It was also shown in two cases that CBCT may not consistently detect narrow buccal and lingual defects with complete accuracy. As expected, PAs underestimated the extension to buccal, lingual, and palatal surfaces in the majority of cases. Additionally, as demonstrated in two of the cases, it posed a difficulty in terms of determining whether mesial and distal defects were joined together by the buccal or lingual/palatal surfaces, or whether they were separate defects.

### 4.3. Angle Measurements

Defect angle is commonly measured on PAs as the angle between the root surface and the defect surface. The limitation of using the reference points CEJ and BD as the root surface [40,41,42] is that root curvature may be present in some cases but not accounted for, which may lead to underestimation of the actual angle between the root surface and the defect wall. Nonetheless, the CEJ remains a reasonably reproducible reference point.

In the previous literature, to our knowledge, only one study has measured the defect angle on CBCT [43]. Their study used the same method as Steffensen and Webert [40] for measurement, but did not mention how the CBCT slice was reconstructed for this measurement and the slice thickness was not reported. Furthermore, whether the views were fixed for the two examiners was not recorded and, therefore, the reproducibility of measurements between the two examiners could not be critically evaluated.

In the present study, an attempt was made to locate the most apical and coronal extents of the defect on CBCT and to bring those points onto the same vertical plane for angle measurement, in order to theoretically obtain an angle comparable to that measured from the PAs. This yielded high reproducibility and agreement between examiners (ICC > 0.9), as well as a high level of agreement between the CBCT and PA angle measurements (ICC = 0.894). CBCT overcomes the difficulty in locating those reference points on a 2-dimensional radiograph, where overlapping or distortion may be present. Furthermore, an infrabony defect is a three-dimensional structure with angles that can be measured in various planes. A different angle can be obtained for other portions of the defect, ranging from apical to coronal and from buccal to lingual/palatal. In this way, CBCT volumetric data provide significantly more information concerning the defect angle than conventional radiographs. As the defect angle is a factor affecting radiographic bone gain and CAL gain for regenerative therapy [44], it may be helpful during treatment planning to be able to visualize the proportion of the defect with an angle that is more favorable for regeneration through the use of CBCT data.

### 4.4. Factors Affecting the Accuracy of PAs and CBCT

The accuracy of both PA and CBCT imaging is affected by inherent limitations. Early lesions with minimal bone mineral loss may not exhibit detectable radiographic changes [8]. PA imaging is affected by the geometric alignment of the image receptor plane, teeth, and the central beam of the X-ray [8], which may have affected the accuracy of the obtained results. In clinical applications, the degree of exposure also affects the visibility of details. As outlined earlier, the interpretation of PA images may be affected by the overlapping of anatomical structures and their two-dimensional nature. The resolution of CBCT is affected by the voxel size [8]. Furthermore, in clinical practice, artifacts and patient movement may influence image quality [8].

### 4.5. The Use of CBCT in Periodontal Assessment and Treatment Planning

Due to the higher radiation exposure and costs involved in CBCT imaging compared to PAs, multiple systematic reviews were still cautious in making recommendations concerning its use in the diagnosis and treatment planning of infrabony defects, suggesting that it should be used in carefully selected cases [10,11]. The effective dose of a set of PAs for the full mouth is approximately 40 μSv [45], while the effective dose of CBCT differs between machines and the protocol used; ideally, it should be between 20 and 100 μSv, which is equivalent to 2–10 panoramic radiographs. Clinical judgment should be used to select cases where further information is required from a CBCT image for diagnosis and treatment planning, and it is crucial to take both diagnostic and clinical benefits and radiation safety into consideration, in alignment with the ALADAIP (As Low as Diagnostically Acceptable being Indication-oriented and Patient-specific) principle [46]. Additionally, a smaller FOV could be used for dose reduction [45].

Therefore, in cases where a more accurate preoperative assessment and treatment planning are desired—such as in the case of more complex defect morphology or when higher surgical difficulty is expected—CBCT could be the method of choice, provided radiation safety principles are adhered to. Periodontal defects should be assessed in patients who undergo CBCT imaging for other purposes, such as implant planning. If the assessment requires only limited coverage of a localized site, a small field of view (FOV) could be used to minimize radiation exposure.

### 4.6. Limitations of the Current Study

#### 4.6.1. Accuracy of Intrasurgical Measurements

As this study used ISs as the gold standard for comparison, the accuracy of ISs would impact the results. Similar to other studies involving intrasurgical measurements, limitations exist that could lead to inaccuracies. The present study used a periodontal probe with markings at 0.5 mm increments, which allowed for more accurate measurements than previous studies [15,32,33,35,36] utilizing probes with markings 1 mm apart. However, probe angulation was limited by the tooth crowns in some cases. An endodontic reamer with a rubber stopper was used in one study [30], and the distance was measured using a digital caliper. However, measurements could only be taken in the facial aspect, as they also reported difficulty in keeping the reamer parallel to the root. Similarly, intrasurgical measurements were obtained with a Williams probe and rubber stop in another study [34], marking the distance to be measured with a digital caliper. More recently, a K-file and a reference stent with grooves that allow for measurements parallel to the root surface were used, and a composite material served as the reference point to be visualized in the CBCT images [31]. The distance between the rubber stopper and the file tip was measured with an endodontic ruler. This method could be considered for future studies, and a digital caliper could be used to calculate the file’s dimensions, rather than a ruler, to further increase the accuracy.

#### 4.6.2. Standardization of Radiographic Examination Protocols

In this study, the CBCT imaging protocol was not standardized to a precise value for all parameters, as some patients already had pre-existing CBCT records before our research began; therefore, there could have been indications for modifying the settings depending on specific scenarios. The samples included data obtained from 2 different CBCT machines. The image resolution also depends on the voxel size used. Nevertheless, the differences were within an acceptable range, and the quality of the images was considered to be diagnostically acceptable by the examiners before being included in the study. A previous study [47] revealed that alveolar bone loss was overestimated when a larger voxel size of 0.4 mm was used, compared to 0.25 mm. Therefore, the present study excluded voxel sizes greater than 0.4 mm to ensure adequate image resolution. Moreover, the CBCT assessment method—including parameters for image reconstruction such as plane orientation and slice thickness—was standardized. Slice thickness or the usage of different views on CBCT for measurements could have an impact on the accuracy of measurements, as one study [23] found a 0.47 mean error when using the 5.2 mm panoramic reconstruction view versus a 0.29 mm mean error when using cross-sections with 0.4 mm thickness. Similar results were demonstrated in [48], where the 5.1 mm panoramic image showed a larger deviation from the gold standard than 0.32 mm cross-sectional slices. On the contrary, no significant differences were found in measurements using 0.25 mm, 0.1 mm, or 3 mm section thickness when compared to intrasurgical measurements [31]. In the present study, all slice thicknesses were standardized to 0.2 mm for higher resolution.

#### 4.6.3. Sample Size

Another limitation is the smaller sample size compared to some studies. Although the sample size met the calculated expectations for primary outcomes, it may have limited the study’s ability to evaluate significant outcomes between different types of infrabony defects or defects with extensions to different root surfaces. Different factors have been found to significantly influence the accuracy, such as the tooth type/location and tooth surface [49]. Future studies can incorporate a broader range of defect types and extensions with a larger sample.

#### 4.6.4. Applicability of the Results

Teeth with metallic restorations, root-filling materials, or dental implants at neighboring sites were excluded in this study to minimize image artifacts. This may reduce the generalizability of the findings to routine clinical settings, where metallic restorations and implants are commonly seen.

## 5. Conclusions

(1) No significant differences were found between CBCT and intrasurgical measurements of infrabony defect depth and width, based on an assessment of CBCT axial slices.
(2) CBCT demonstrated high agreement with intrasurgical observations in all defect characteristics, including depth, width, defect type (categorized as predominantly one-wall, two-wall, or three-wall), and defect extension (specified according to the involved root surfaces).
(3) CBCT showed higher accuracy than PAs in measuring defect width when compared to intrasurgical measurement as the gold standard, as well as higher agreement in the determination of defect type and extension than PAs.

## Figures and Tables

**Table 1:** ICC between intrasurgical and CBCT/PA measurements for depth and width.

	Depth		Width	
	ICC	95% CI	ICC	95% CI
IS–CBCT	0.938	0.869–0.972	0.923	0.838–0.965
IS–PA	0.790	0.588–0.900	0.704	0.410–0.860

**Table 2:** Paired *t*-test results for mean differences in measurements between CBCT/PA and intrasurgical measurements.

		Mean Difference ± SD	95% CI	*p*-Value
CBCT				
	Depth (mm)	−0.11 ± 0.49	−0.31–0.09	0.274
	Width (mm)	−0.07 ± 0.41	−0.23–0.01	0.420
PA				
	Depth (mm)	0.15 ± 0.95	−0.23–0.53	0.427
	Width (mm)	−0.36 ± 0.73	−0.65–−0.06	0.019 *

* *p* < 0.05.

**Table 3:** Agreement between intrasurgical and CBCT or PA observations for defect type and extension (*n* = 26).

	IS–CBCT		IS–PA	
	Cohen’s Kappa	% of Total Agreement	Cohen’s Kappa	% of Total Agreement
Defect Type	0.819	88 (*n* = 23)	0.214	46 * (*n* = 12)
Defect Extension	0.855	88 (*n* = 23)	0.381	54 ^†^ (*n* = 14)

* *p* = 0.007 for % of total agreement with PAs compared to % of total agreement with CBCT. ^†^ *p* = 0.012 for % of total agreement with PAs compared to % of total agreement with CBCT.

## Data Availability

The original contributions presented in this study are included in the article. Further inquiries can be directed to the corresponding author.

## Appendix A

**Table A1:** Frequency distribution (by defect) (*n* = 26).

		*n*	Percentage
Gender			
	Male	9	35%
	Female	17	65%
CBCT			
	Planmeca	18	69%
	Morita	8	31%
Voxel Size			
	125 µm	14	54%
	200 µm	12	46%
Tooth Type			
	Anterior	6	23%
	Premolar	18	69%
	Molar	2	8%
Surgery Performed			
	Access Flap	1	4%
	OFD with osseous	9	35%
	Regeneration	16	61%
Flap Design			
	M-MIST	3	11%
	MIST	8	31%
	Extended	6	23%
	Resective	9	35%
Defect Type (Predominance)			
	One-wall	6	23%
	Two-wall	9	35%
	Three-wall	11	42%
Defect Extension			
	Single surface	14	54%
	Multiple surfaces	12	46%

**Table A2:** Descriptive statistics: mean, minimum, maximum, and standard deviation for linear measurements and defect angle.

	*n*	Minimum	Maximum	Mean	S.D.
**IS**	26				
Depth		1.0	8.00	4.11	1.54
Width		1.5	6.0	2.96	1.14
**CBCT**	26				
Depth		1.2	7.2	4.01	1.26
Width		1.4	5.4	2.90	0.91
Angle		18.1	48.0	32.16	8.03
**PA**	26				
Depth		1.9	7.1	4.27	1.37
Width		1.1	4.7	2.60	0.86
Angle		18.2	50.4	31.06	9.30

**Table A3:** Intra- and inter-examiner reliability for depth, width, and angle measurements with CBCT and PAs.

	Examiner 1		Examiner 2		Inter-Examiner	
	ICC	95% CI	ICC	95% CI	ICC	95% CI
**CBCT**						
Depth	1.000	---	1.000	---	0.922	0.876–0.964
Width	0.972	0.938–0.987	0.960	0.914–0.982	0.905	0.802–0.956
Angle	0.926	0.843–0.966	0.918	0.826–0.963	0.915	0.821–0.961
**PA**						
Depth	0.990	0.977–0.995	0.987	0.972–0.994	0.925	0.841–0.966
Width	0.980	0.951–0.991	0.965	0.924–0.984	0.917	0.904–0.981
Angle	0.940	0.865–0.973	0.959	0.911–0.981	0.889	0.770–0.949

**Table A4:** Inter-examiner reliability for defect type and defect extension.

	Cohen’s Kappa		% of Total Agreement	
	CBCT	PA	CBCT	PA
Defect Type	0.876	0.631	92.3	76.9
Defect Extension	0.951	0.745	96.2	84.6
